# A study on and adsorption mechanism of ammonium nitrogen by modified corn straw biochar

**DOI:** 10.1098/rsos.221535

**Published:** 2023-02-08

**Authors:** Sinan Wang, Hongshuo Zhao, Jinhua Liu, Xue Wang, Jiahao Li, Enping Shi, Ci Wang, Jingmin Yang, Zhongqing Zhang

**Affiliations:** ^1^ College of Resources and Environment, Jilin Agricultural University/Key Laboratory of Sustainable Utilization of Soil Resources in Commodity Grain Base of Jilin Province, Key Laboratory of Straw Comprehensive Utilization and Black Soil Conservation, Ministry of Education, Changchun 130118, People's Republic of China; ^2^ College of Environmental Science and Engineering, Tongji University, Shanghai, People's Republic of China; ^3^ Jilin Provincial Agro-Tech Extension Station, Changchun, People's Republic of China

**Keywords:** corn straw, biochar, ammonium nitrogen

## Abstract

Using corn stover as raw material, the adsorption mechanism of ammonium nitrogen by biochar prepared by different modification methods was studied. The biochar was characterized by Fourier transform infrared spectroscopy, surface-area analysis and scanning electron microscopy. The results showed that the adsorption of ${\mathrm{NH}}_{4}^{+}-N$ by different modified biochars confirmed the quasi-second-order kinetic equation (*R*^2^ > 0.95, *p* ≤ 0.05), the adsorption isotherms of the Langmuir equation (*R*^2^ ≥ 0.96, *p* ≤ 0.05). Δ*G*^θ^ < 0, Δ*H*^θ^ > 0 indicated that the adsorption of ${\mathrm{NH}}_{4}^{+}-N$ by different modified biochars was a spontaneous endothermic reaction. With the increase in adsorption temperature, the adsorption capacity of biochar to ammonium nitrogen increased gradually. The adsorption was monolayer adsorption and was controlled by a fast reaction. Both KOH and FeCl_3_ modified biochars significantly improved the adsorption capacity of ${\mathrm{NH}}_{4}^{+}-N$, and the adsorption mechanism was different. The adsorption capacity of ${\mathrm{NH}}_{4}^{+}-N$ by FeCl_3_ modified biochars mainly increased the specific surface area and micropore volume. The adsorption of ammonium nitrogen after KOH modification primarily depended on the wealthy oxygen-containing functional groups. The adsorption effect of ammonium nitrogen modified by KOH was better than that of biochar modified by FeCl_3_.

## 1. Introduction

China is a large agricultural country. There are many crops and large amounts of crop straw every year [1]. Among them, corn straw accounts for 41.92% of the total output of crop straw [2]. The traditional treatment methods of corn straw mainly include buring [3], returning to the field [4], silage [5], pyrolysis carbonization [6], hydrolysis carbonization and so on [7]. Among them, the biocharization process is simple, the product cost is low, the processing equipment is simple, and it can be applied on a large scale [8]. Biocharization is a hydrothermal reaction in which biomass and water are mixed in a fixed proportion and put into an autoclave at a certain temperature (180°C−350°C), reaction time and atmospheric pressure. Its target product is a lignite-like solid product [9]. As an economical and efficient adsorption material, carbon synthesized by biocharization has attracted extensive attention in recent years [10]. The price of modified biochar is close to half that of activated carbon, and the adsorption capacity is equivalent to that of activated carbon [11]. The adsorption capacity is significantly higher than that of traditional adsorbents [12]. The adsorption of atrazine in water by corn straw hydrothermal charcoal was studied. Many studies have shown that biochar can be used as an adsorbent to remove toxic elements and antibiotics in soil and water, reduce greenhouse gas emissions and restore nutrients [13–15]. Therefore, the preparation of biochar provides an effective means for the comprehensive utilization of corn straw.

In recent years, with large amounts of fertilizer use, ammonium nitrogen enters the water body through leaching and surface runoff has increased, resulting in more serious water eutrophication [16]. There are many methods to reduce the concentration of ammonium nitrogen in the water, including the guanite precipitation method [17], ammonia stripping method [18] and adsorption method [19]. Among them, the adsorption method is widely used to recover high concentration of ammonium nitrogen due to its simplicity. Ammonium nitrogen adsorbents mainly include zeolite [20], activated carbon [21], biochar [18] and kaolinites [22]. Biochar can effectively capture and adsorb ammonium nitrogen from water bodies. It is an environmentally friendly and low-cost potential alternative material. It is widely used to remove ammonium nitrogen from eutrophic water bodies [23]. However, due to the low specific surface area, porosity and less oxygen-containing surface functional groups of biochar prepared by ordinary methods [14], its ability to adsorb ammonium nitrogen is significantly lower than that of biochar adsorbent prepared by pyrolysis [11]. Therefore, it is necessary to activate and modify biochar and increase the content of oxygen-containing functional groups to improve its adsorption capacity [24].

Many studies have shown that it is difficult to obtain high-quality biochar adsorbents only by adjusting hydrothermal environmental parameters (such as reaction temperature, pressure and residence time) [25]. Therefore, various modification methods need to be adopted to improve the adsorption performance of biochar, such as clay mineral [26], acid-base activation [27], metal salt impregnation [6], ball milling [28] and so on. Among them, Fe modification significantly enhances the specific surface area and micropore volume of biochar [29]; Wang *et al*. [30] concluded that the adsorption performance of iron-modified biochar prepared by the acid impregnation modification method for ammonium nitrogen was 24.1% higher than unmodified biochar; Zhi *et al*. [31] showed that the maximum nitrogen adsorption capacity of iron-modified peanut biochar was 41.58 mg g^−1^. This method was prepared by wet impregnation pyrolysis and the adsorption performance was significantly improved. Alkali activation can enhance the electrostatic interaction and ion exchange on the surface of biochar [32], which can effectively improve the adsorption capacity of ammonium nitrogen [33]; Zhang *et al*. [34] studied the adsorption of ammonium nitrogen by KOH-modified corn stover, and the results showed that the modified biochar had a better effect compared with the modification, the oxygen-containing functional groups become more abundant, the specific surface area is almost doubled and the adsorption capacity is significantly improved. The potassium hydroxide activated biochar is a promising ammonium composite.

There are many studies on the preparation of corn straw biochar using the pyrolysis method as an adsorbent [35]. Still, there are few reports on the practice of corn straw biochar by the hydrothermal method as adsorbent [34]. In this paper, corn straw biochar was prepared by using corn stalk as a raw material and modified by potassium hydroxide and ferric chloride. The adsorption kinetics and adsorption thermodynamics of ammonium nitrogen in water were studied, and the structural characteristics of different modified corn straw biochar before and after adsorption were compared and analysed. At the same time, the adsorption mechanism of ammonium nitrogen on corn stalk modified biochar was discussed, which provided theoretical support for the treatment of an eutrophic water body.

## 2. Material and methods

### 2.1. Chemicals and equipments

Chemicals are all analytically pure and purchased from China National Pharmaceutical Group, including (NH_4_)_2_SO_4_.5H_2_O, C_3_Cl_2_N_3_NaO_3_.2H_2_O, NaOH, Na_2_[Fe(CN)_5_NO].2H_2_O, NaC_7_H_5_O_3_ and C_6_H_5_Na_3_O_7_.2H_2_O.

Equipment includes a scanning electron microscope (JSM-7900F, Japan), infrared spectrum scanner (FTIR-IRAffinity-1 s, Japan), constant temperature oscillator (SHA-2, Jintan Ruihua Instrument Co., Ltd.), electrothermal constant temperature drying oven (202-3AB, Tianjin test Instrument Co., Ltd.), magnetic stirrer (DJ-1, Jiangsu Scientific Analysis Instrument Co., Ltd.) and continuous flow injection analyser (SKALAR SAN++, Netherlands).

### 2.2. Preparation of biochar

In this study, the corn straw was collected from the experimental field of Jilin Agricultural University. The corn straw was first dried at 70 grounded, and passed the 70 target standard sieve. After this, accurately weighted 8.0 g (HC) corn straw, and another 8.0 g was added into 2.0 g ferric chloride (HD) solid, mixed with 100 ml deionized water, stirred at room temperature for 30 min, transferred to a reaction kettle, placed in an electric constant temperature drying oven, kept at 240°C for 24 h, cooled to room temperature, then taken out and washed with deionized water to neutrality. After drying at 60°C for 8 h. HC was mixed with 3 mol l^−1^ KOH at a solid−liquid ratio of 1 : 10, shaken at 25°C and heated at 200 r min^−1^ for 3 h to obtain activated biochar, which was washed with deionized water to neutrality, and dried in an oven at 105°C for 24 h to obtain biochar labelled HCK.

### 2.3. Characterization of biochar

The specific surface area and pore size distribution of three kinds of corn stalk hydrothermal charcoal were measured by static nitrogen adsorption instrument (3H-2000PS1 model, Beside Instrument Technology (Beijing Co., Ltd.)). The BET model was selected for specific surface area measurement, and the DFT model was selected for micropore volume, total pore volume and average pore size calculation. HC, HD, HCK and the apparent morphology of biochar of corn stalks were measured and analysed by electron scanning microscope. Infrared spectra of HC, HD and HCK biochar of corn stalks were measured and analysed by infrared scanner before and after the adsorption of ammonium nitrogen.

### 2.4. Adsorption test

#### 2.4.1. Adsorption kinetics test

The adsorption kinetics test was carried out by batch equilibrium method. Three kinds of carbon (0.05 g) and ammonium nitrogen (100 mg l^−1^) aqueous solutions (25 ml) were weighed and put into a 50 ml centrifuge tube, respectively, and oscillated at 25°C, 35°C and 45°C. Samples were taken out at 5, 10, 30, 60, 120, 360 and 720 min, respectively. After filtration, the concentration of ammonium nitrogen in filtrate was determined, the adsorption capacity of ammonium nitrogen by biochar was calculated, the adsorption kinetic curve of ammonium nitrogen was drawn, the adsorption kinetic equation was fitted, and the kinetic parameters were calculated.

#### 2.4.2. Adsorption thermodynamics test

The adsorption thermodynamic test adopts the batch equilibrium method, three kinds of carbons (0.05 g) were weighed, and 25 ml of aqueous solution with ammonium nitrogen concentrations of 0, 10, 30, 50, 70, 100 and 200 mg l^−1^ were put into a 50 ml centrifuge tube, and the samples were taken out by shaking at 25°C, 35°C and 45°C for 24 h. After filtration, the concentration of ammonium nitrogen in the filtrate was measured, and the adsorption capacity of ammonium nitrogen by biochar was calculated. The isothermal adsorption curve of ammonium nitrogen was drawn, the isothermal adsorption equation was fitted and the thermodynamic parameters were calculated.

### 2.5. Data processing

#### 2.5.1. Calculation of adsorption capacity

2.1 $$Q=\frac{C_{0}-C}{M\times V};\;$$

where *Q* is the adsorption amount of atrazine (mg g^−1^); *C*_0_ and *C* are the initial and equilibrium concentrations of atrazine (mg l^−1^), respectively; *V* is the volume of adsorption liquid (*L*) and *M* is the mass of the adsorbent (*g*).

#### 2.5.2. Adsorption kinetic model

2.2 $$\mathrm{quasi}-\mathrm{first}-\mathrm{order}\;\mathrm{model}:\;\ln(q_{e}-q_{t})=\frac{\ln\;q_{e1}-k_{1}t}{2.303}$$

2.3 $$\mathrm{quasi}-\mathrm{second}-\mathrm{order}\;\mathrm{model}:\;\frac{t}{q_{t}}=\frac{1}{k_{2}q_{e}^{2}}+\frac{t}{q_{e}},$$

where *q_e_* and *q_t_* are the adsorption capacity of atrazine by biochar at equilibrium and time *t* (mg g^−1^), respectively; *k*_1_ is the constant of the quasi-first-order adsorption model (g mg min^−1^); *k*_2_ is the quasi-second-order adsorption model constant (g mg min^−1^).

#### 2.5.3. Adsorption thermodynamics model

2.4 $$\mathrm{Langmuir}:\;\frac{C_{e}}{q_{e}}=\frac{C_{e}}{q_{m}}+\frac{1}{q_{m}k_{L}}$$

and

2.5 $$\mathrm{Freundlich}:\;lgq_{e}=nlgC_{e}+lgk_{F},$$

where *q_e_* is the adsorption capacity (mg kg^−1^), *C_e_* is the concentration of adsorption equilibrium solution (mg l^−1^), *q_m_* is the maximum adsorption capacity (mg kg^−1^), and *K_L_* and *K_F_* are the adsorption constants of Langmuir and Freundlich, respectively.

#### 2.5.4. Van't Hoff thermodynamic parameters

2.6 $$\Delta G=-\mathrm{RT}\;\ln k_{L}$$

and

2.7 $$\Delta S=\frac{\Delta H-\Delta G}{T},$$

where Δ*G^θ^* is the Gibbs free energy; R (8.314 J K mol^−1^) is the gas constant; *T* is the absolute temperature; *K_L_* is the adsorption equilibrium constant of the Langmuir model; Δ*H^θ^* is the change in enthalpy; Δ*S^θ^* is the change in entropy. The values of Δ*H^θ^* and Δ*S^θ^* were calculated according to the slope and intercept of the plot of lnK_L_ versus 1/*t*.

## 3. Results and discussion

### 3.1. Analysis of specific surface area and pore structure

During the preparation of corn straw biochar, the internal structure of corn straw was destroyed by dehydration and pyrolysis of hemicellulose [36]. With the increase in pyrolysis temperature, the porosity of carbon-rich materials gradually increased. When the temperature increased to 240°C, the pyrolysed carbon-rich particles carbonized, a large amount of energy rushed out of the pores, and the pore distribution became disordered. The continuous pyrolysis of lignin made the pore wall thinner, and at the same time produced a large number of micropore structures, forming biochar microspheres with rich void structures [37]. The specific surface area, pore volume and pore size of HC, HD and HCK corn stalk biochar microspheres were determined by the BET method and BJH adsorption/desorption method.

The analysis results of specific surface area of corn straw biochars was shown in table 1. HCK and HD modified biochar significantly increased the specific surface area of corn straw biochar microspheres by 61.84 and 33.33%, the total pore volume by 31.6 and 21.47%, the micropore volume by 7.1 and 4.8% and the average pore size by 29.53 and 13.19%. This was due to the fact that the adoption of KOH and FeCl_3_ improved the pore properties of biochar [38]. Through modification, small pores combined to form large pores, resulting in an increase in specific surface area, average pore size and micropore volume [39].

**Table 1.** Specific surface area and pore structure of corn straw modified biochar.

biochar	specific surface area (m^2^ g^−1^)	total pore volume (cm^3^ g^−1^)	micropore volume (cm^3^ g^−1^)	average aperture (nm)
HC	15.12	0.1826	0.0042	49.07
HD	20.16	0.2218	0.0044	55.54
HCK	24.47	0.2403	0.0045	63.56

### 3.2. Electron scanning microscope before and after adsorption

**Figure 1.**
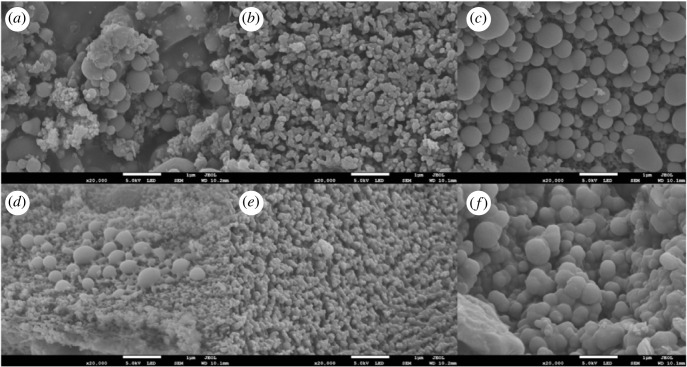
Scanning electron microscope of corn straw modified nano biochar microspheres; (*a*), (*b*) and (*c*) are HC, HD and HCK, respectively; (*d*), (*e*) and (*f*) are HC, HD and HCK after adsorption of ammonium nitrogen.

At 240°C, hemicellulose and cellulose in corn stalk were polymerized, dehydrated and carbonized rapidly, and biomass was decomposed to form nano-carbon microspheres [40]. The microstructure of three biochars before and after the adsorption of ammonium nitrogen was characterized by an electron scanning microscope (figure 1). Before adsorbing ammonium nitrogen, photo (*a*) shows the porous structure and carbon spheres of corn straw biochar, but some pores will be covered and blocked by impurities, photo (*b*) shows a raspberry-like structure formed on the surface with many fine pore structures. After KOH modification and activation in photo (*c*), some impurities blocking the pore structure are decomposed, or volatilized [41]. We also see that in photo (*c*), more obvious pore structure and microsphere structure appear, and monodisperse carbon microspheres appear, indicating that the pore structure of biochar is increased through the modification of FeCl_3_ and KOH, which is consistent with the measured specific surface area by Nguyen *et al*. [42].

After adsorption, the surface of biochar scanning microscopes was shown in the photos (*d*), (*e*) and (*f*). The photos illustrated that the surface was attached with particles or powders, and the pores are filled, the structure of carbon microspheres was reduced, and the surface was rougher than that before adsorption. Photo (*a*) shows more regular, smooth and dense surface than photo (*d*) with a small amount of carbon microsphere structure. Photo (*b*) shows filled surface pores, and more decreased structure of carbon microspheres compared with photo (*d*). Photo (*c*) shows no obvious carbon microsphere particle profile, irregular shape, and filled pore between the carbon microsphere structure compared to photo (*e*). The surface structures of (*e*) and (*f*) are still more than that of (*d*).

### 3.3. Fourier infrared spectra before and after adsorption

**Figure 2.**
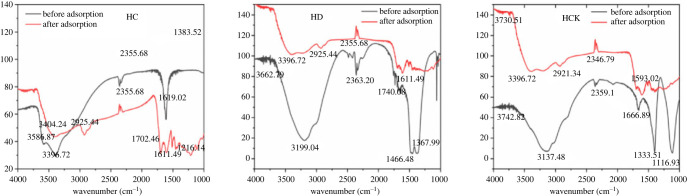
FT-IR analysis of corn straw modified biochar.

Figure 2 shows that three biochars had absorption peaks in the range of 3100–3500 cm^−1^, but the peaks of HD and HCK were larger than HC. This absorption peak was mainly caused by the −OH stretching vibration of intermolecular hydrogen bond association [43]. After adsorption, the peak value decreased, the peak position shifted and the peak shape narrowed, indicating that the hydroxyl group (−OH) on the surface of biochar participated in the adsorption of ammonium nitrogen. The absorption peaks of three biochars appeared at 2300–2400 cm^−1^, which was generated by the stretching vibration of methyl (−CH_3_) and methylene (−CH_2_) in aliphatic hydrocarbons or cycloalkanes [44]. There was no significant change after adsorption. The absorption peaks of three biochars ranged between 1610–1667 cm^−1^ with the stretching vibration of aromatic cyclocarbonyl (−C=O) [45]. After the adsorption of the three biochars, the wave peaks were significantly narrowed, indicating that the surface of biochars −C=O was involved in the adsorption. The modified HD and HCK exhibited stretching vibration with the absorption peak in alcohol or phenol (−C–O) at the wavenumber of 1368–1467 cm^−1^ [46]. After adsorption, the intensity of absorption peaks became weaker and the peaks became narrower than that before adsorption, indicating that this group also participated in the adsorption of ammonium nitrogen. The absorption peak of HCK at 1116 cm^−1^ was the bending vibration of alcohol hydroxyl (−OH) and the stretching vibration of ether (−O−) [47]. After adsorption, the absorption peak intensity is weaker and the wave peak is narrower than that before adsorption, which indicates that this group also participates in the adsorption of ammonium nitrogen. Compared with the unmodified biochar, the modified biochar had more functional groups to participate in the adsorption process, the absorption peaks of each functional group were weakened after adsorption, and new chemical bonds were formed in the adsorption process, which reduced the peak intensity.

### 3.4. Adsorption kinetic curves of ammonium nitrogen on different modified biochars

**Figure 3.**
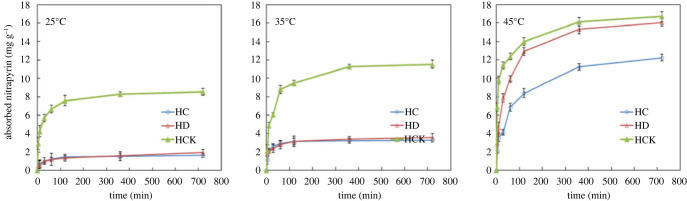
Adsorption kinetics of ammonia nitrogen by corn straw modified biochar.

The adsorption of ammonium nitrogen by biochar can be divided into two stages: rapid adsorption in the first stage and slow adsorption in the second stage [34]. The adsorption kinetic curves of ammonium nitrogen by different modified biochars (figure 3) showed that a fast adsorption stage was from 0 to 130 min and a slow adsorption stage was from 130 to 360 min. During the late stage, the adsorption curve tends to be stable, and the adsorption rate is slow. After 360 min, the adsorption capacity reaches equilibrium, and the adsorption of ammonium nitrogen reaches the maximum saturated adsorption. With the increase of time, the adsorption capacity does not change significantly.

The adsorption capacity of biochar is HCK > HD > HC, and the adsorption capacity of the three kinds of biochar increases with the increase of adsorption temperature. At 25°C and 35°C, the adsorption capacity of ammonium nitrogen on KOH modified biochar is significantly higher than that of FeCl_3_ altered and unmodified biochar. At 45°C, the adsorption capacity of three biochars for ammonium nitrogen increased obviously, but the effect of KOH and FeCl_3_ modified carbons greatly exceeded unmodified biochars. The results show that KOH modification can significantly improve the adsorption performance of biochar at any temperature, and FeCl_3_ improvement can improve the adsorption performance of biochar only at high temperatures.

To explore the kinetic adsorption mechanism of ammonium nitrogen by modified corn straw biochar, the adsorption data were fitted and analysed by quasi-first-order kinetic equation and quasi-second-order kinetic equation (table 2), the *R*^2^ ranged between 0.89–0.97 in the fitting parameters of the quasi-first-order kinetic model, and the *R*^2^ ranged between 0.95–0.99 in the fitting parameters of the quasi-second-order kinetic model, indicating that both models were fitted significantly while the reasonable degree of the quasi-second-order kinetic model is higher than the quasi-first-order model. Therefore, the quasi-second-order kinetic equation can better describe the kinetic adsorption process of ammonium nitrogen by biochar. Based on the *K*_2_ value in the quasi-second-order kinetic model, the adsorption of ammonium nitrogen by the three modified biochars is mainly controlled by the rapid reaction [48]. The equilibrium adsorption capacity (*q_e_*) of HCK for ammonium nitrogen was significantly higher than that of HC and HD; the equilibrium adsorption capacity of ammonium nitrogen was more than doubled. The results showed that KOH modification significantly improved the adsorption of ammonium nitrogen by corn straw biochar.

**Table 2.** Kinetic parameters of ammonia nitrogen adsorption by corn straw modified biochar.

handle	sample	*q_e_*_(_mg g^−1^)	quasi-first-order			quasi-second-order		
			*Q*_1_ (mg g^−1^)	*K*_1_	*R*^2^	*Q*_2_ (mg·g^−1^)	*K*_2_	*R*^2^
298	HC	1.64	1.47	0.0545	0.90	1.59	0.0512	0.96
	HD	1.96	1.62	0.0351	0.89	1.78	0.0270	0.95
	HCK	8.53	7.70	0.0689	0.94	8.35	0.0111	0.98
308	HC	3.29	3.08	0.0987	0.97	3.30	0.0408	0.98
	HD	3.54	3.06	0.1282	0.89	3.29	0.0509	0.96
	HCK	11.54	10.71	0.0338	0.94	11.65	0.0043	0.98
318	HC	12.22	11.43	0.0152	0.94	12.64	0.0016	0.96
	HD	16.06	15.10	0.0221	0.96	16.47	0.0019	0.99
	HCK	16.70	14.31	0.1114	0.90	15.53	0.0088	0.96

### 3.5. Adsorption isotherms of ammonium nitrogen on different modified biochars

**Figure 4.**
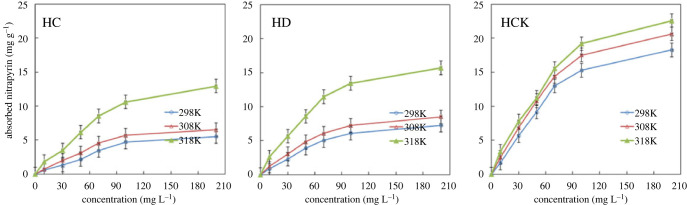
Adsorption isotherm of corn straw modified biochar.

Figure 4 shows the adsorption isotherms of ammonium nitrogen by three types of biochars at 25°C (298 K), 35°C (308 K) and 45°C (318 K) with the fitted curves. With the increase of ammonium nitrogen concentration in solution, the adsorption amount of ammonium nitrogen by these three kinds of biochars gradually increases. With the increase of temperature, the adsorption capacity of ammonium nitrogen by biochar increases, which indicates that the increase of temperature and ammonium nitrogen concentration can promote the adsorption of ammonium nitrogen by biochar. At any temperature, the adsorption capacity of ammonium nitrogen by KOH modified biochar is significantly greater than that of FeCl_3_ modified and unmodified biochar.

Table 3 shows the fitted isothermal carbon equations for ammonium nitrogen adsorption under the modified biochars of HC, HD and HCK. The adsorption isothermal process of ammonium nitrogen by HC, HD and HCK was significantly fitted by the Langmuir model (*R*^2^ ≥ 0.96), which demonstrates that the ammonium nitrogen adsorption is suited to monolayer sorption and mainly dominated by chemisorption [33,49]. However, because a relatively high *R*^2^ of 0.94 was obtained using the Freundlich model, physical sorption may also have occurred [34]. With the increase of temperature, the reaction rate constants (*K_L_* and *K_F_*) increased, indicating that increasing temperature accelerated the adsorption rate of ammonium nitrogen by HC, HD and HDK.

**Table 3.** Parameters of isothermal fitting equations for ammonia nitrogen adsorption by corn straw modified biochar.

sample	temperature *K*	Langmuir			Freundlich		
		*q_m_*/(mg g^−1^)	*K_L_*/(l mg^−1^)	*R*^2^	*n*	*K_F_*/[mg g^−1^(mg l^−1^)^−1/n^]	*R*^2^
HC	298	10.05	0.0068	0.96	1.58	0.2103	0.94
	308	10.25	0.0101	0.97	1.80	0.3756	0.94
	318	20.81	0.0090	0.98	1.75	0.6702	0.96
HD	298	10.97	0.0110	0.98	1.86	0.4558	0.95
	308	12.12	0.0132	0.99	2.00	0.6502	0.96
	318	22.24	0.0135	0.99	2.03	1.2472	0.95
HCK	298	28.32	0.0102	0.98	1.80	1.0413	0.94
	308	30.91	0.0113	0.98	1.88	1.3275	0.95
	318	33.65	0.0113	0.99	1.90	1.4964	0.96

Studies show that the adsorption capacity of activated carbon for ammonia nitrogen is about 0.20 mg g^−1^. [21]. However, the maximum adsorption capacities of HC, HD and HCK for ammonium nitrogen at 45°C were 12.99 mg g^−1^, 15.71 mg g^−1^ and 22.61 mg g^−1^, respectively, which is much larger than the adsorption capacity of activated carbon for ammonium nitrogen. The maximum adsorption capacity of HCK for ammonium nitrogen was significantly higher than that of HC and HD, indicating that the HCK modified by KOH substantially improved the adsorption capacity of ammonium nitrogen.

According to the linear slope and intercept obtained by the Van't Hoff equation, Δ*G^θ^* and Δ*H^θ^* were calculated, and then Δ*S^θ^* values were obtained. The results (table 4) showed Δ*G^θ^* < 0 and Δ*H^θ^* > 0, indicating that the adsorption of ammonium nitrogen by HC, HD and HCK results from spontaneous endothermic reaction. The negative Δ*G* value indicated that ammonium nitrogen spontaneously moved from the solution to the surface of corn straw biochar microspheres during the adsorption reaction.

**Table 4.** Thermodynamic parameters of adsorption of ammonia nitrogen by corn stover modified hydrothermal charcoal.

sample	temperature *K*	Δ*Gθ*/(kJ mol^−1^)	Δ*Sθ*/(J·(mol K)^−1^)	Δ*Hθ*/(kJ mol^−1^)
HC	298	−4.75	0.0398	6.6575
	308	−5.92		
	318	−5.81		
HD	298	−5.94	0.0328	3.5237
	308	−6.61		
	318	−6.88		
HCK	298	−5.75	0.0229	0.8762
	308	−6.21		
	318	−6.41		

The Δ*G* value decreased with the increase of temperature indicating that the increased temperature increases the spontaneity of the reaction. It is generally believed that ΔG*^θ^* physical adsorption was in the range of −20∼0 KJ mol^−1^, while chemical adsorption was in the range of −800∼40 KJ mol^−1^ [50]. Adsorption kinetics and isothermal analysis showed that the adsorption process was mainly a chemical reaction, but the *ΔG* value, concluded that the adsorption process was mainly a physical one. One possible explanation for this difference is that chemical and physical reactions co-occur in the adsorption process. This is consistent with the research results of [34]. The Δ*S* value was positive, indicating that the reaction was related to the increased entropy, and the disorder degree at the solid–liquid interface increases during the adsorption process [33].

### 3.6. Study on adsorption mechanism of ammonium nitrogen by biochar

Some studies proposed that the surface mechanism of biochar mainly included electrostatic attraction, ion exchange, ligand exchange and hydrogen bond [51]. The results showed that the biochars modified by FeCl_3_ and KOH had a large number of micropores and a larger specific surface area compared with the unmodified biochars, which was consistent with the measured data of the specific surface area of the biochars modified by FeCl_3_ and KOH. The results of infrared spectroscopy showed that the modified biochar increased the active functional groups [52], which increased the adsorption capacity of ammonium nitrogen. The adsorption performance of ammonium nitrogen was in the order of KOH modified > FeCl_3_ modified > unmodified. The biochar modified by metal salt had good electrostatic attraction, precipitation and anion exchange capacity, which improved its adsorption capacity [6]. The specific surface area and micropore volume of biochar were significantly increased by Fe modification [29]. Compared with unmodified biochar, FeCl_3_ modified biochar has more different functional groups and more chemical bonds, which enhances the chemisorption ability of biochar [53].

**Figure 5.**
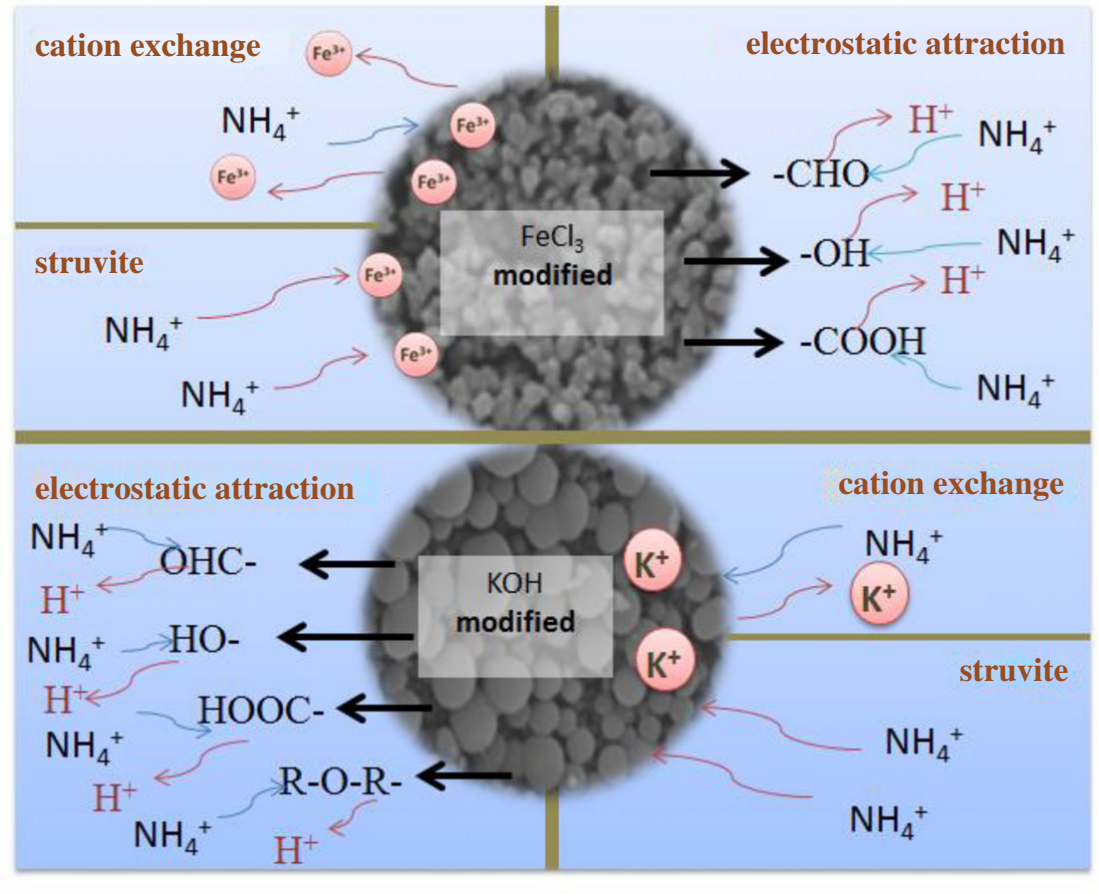
Adsorption mechanism.

The adsorption mechanism is shown in figure 5. It is generally considered that the modification of biochar by KOH has gone through two processes: solid-solid reaction process and solid–liquid reaction process, including potassium ions in KOH changing into potassium atoms, carbon atoms changing into carbon dioxide and carbonate and forming other active intermediates, improving the pore structure of biochar to increase the specific surface area [53], which is an important factor to increase the adsorption capacity of ammonium nitrogen by KOH modified biochar. The role of oxygen-containing functional groups of biochar activated by KOH [27] and ion exchange is the most crucial factor in the adsorption process of ammonium nitrogen [34]. Meanwhile, compared with previous studies on the adsorption of ammonium nitrogen by biochar [14], KOH modified biochar had good adsorption performance.

## 4. Conclusion

(1) The adsorption behaviour of three kinds of biochars for ammonium nitrogen was fitted by different adsorption kinetic models. The fitted models showed more significant *R*^2^ values (=0.9448–0.9928) in the quasi-second-order kinetic model than the quasi-first-order kinetic model (*R*^2^ = 0.8980∼0.9916), indicating that the adsorption process was more in line with the quasi-second-order kinetic model than the quasi-first-order kinetic model.
(2) The results of isothermal adsorption experiments showed that the Langmuir equation could better describe the adsorption process well among three biochars for ammonium nitrogen, mainly monolayer adsorption. The adsorption thermodynamic process Δ*G^θ^* < 0, Δ*H^θ^* > 0, Δ*S* > 0 indicated that adsorption was a spontaneous, endothermic and disordered process. The adsorption process was mainly dominated by chemisorption.
(3) There were abundant oxygen-containing functional groups on the surface of the three biochars, among which –C=O and –C–O functional groups mainly participated in the adsorption process, and the adsorption was monolayer adsorption and controlled by rapid reaction. Both KOH modified and FeCl_3_ modified biochars significantly improved the adsorption capacity of ammonium nitrogen. The adsorption mechanism was different. FeCl_3_ modified biochar mainly increased the specific surface area and micropore volume of biochar; the adsorption of ammonium nitrogen after KOH modification mostly depended on abundant oxygen-containing functional groups. The adsorption effect of ammonium nitrogen after KOH modification was better than that of FeCl_3_ modified biochar.

## Data Availability

Data have been submitted as supplementary materials [54].
